# Open Science at *PLOS Pathogens*

**DOI:** 10.1371/journal.ppat.1011776

**Published:** 2023-11-30

**Authors:** Lauren Cadwallader, Kasturi Haldar, Rebecca Kirk, Neil A. Mabbott, Michael H. Malim

**Affiliations:** 1 PLOS, Cambridge, United Kingdom; 2 Editor-in-Chief, *PLOS Pathogens*, PLOS, United Kingdom; 3 University of Notre Dame, Notre Dame, Indiana, United States of America; 4 The Roslin Institute & Royal (Dick) School of Veterinary Studies, University of Edinburgh, Midlothian, United Kingdom; 5 King’s College London, London, United Kingdom

The pathogens research community has driven, and benefited from, Open Science. As a leader in the field, *PLOS Pathogens* strives to advance community-rooted adoption of practices that enable transparency, rapid communication, reproducibility, and trust in research, and help to transform research globally. A key vision of *PLOS Pathogens* is to facilitate widespread adoption of Open Science to accelerate and improve research that is rigorous and trustworthy and to ensure its meaningful impact on lives.

## The importance of Open Science

**Fig 1:**
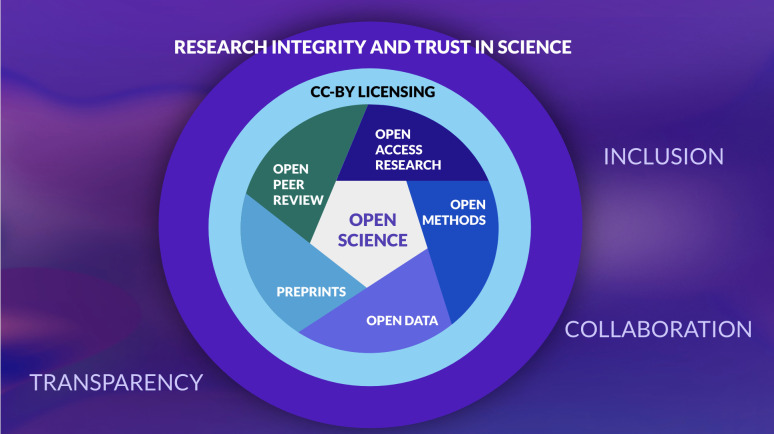
The interrelated parts of Open Science.

Open Science—the public, unrestricted sharing of all components of research—can improve science in many ways and has benefits for a wide range of stakeholders. Some elements of Open Science, such as Open Access to publications, advance equity to ensure research findings can be accessed by everyone with a vested interest in the science. Other elements, such as resource and data sharing, enable researchers to reuse and build on existing science. What all elements have in common is the ability to increase trust, reproducibility, and transparency in science (Fig 1). These attributes are essential not only for the scientific community but also for policymakers, funders, practitioners, and the public. Open Science is rooted in the principles of fairness, justice, and equity and thus enables and empowers global and diverse communities to participate in and contribute to more efficient science [1].

Open Science has long been practiced by the pathogens research community, given its importance in the context of the challenges we seek to address, and the collaborative nature and potential impacts our research can have on the lives of people across the globe. *PLOS Pathogens* was founded with a philosophy based on open, high-quality, rigorous science, initially focusing on Open Access publication and diversifying the ways scientists communicate with the community and public (for example, see [2]) and more recently experimenting with ways to enhance data sharing [3]. We have highlighted the importance of Open Science to the community and discipline since the inception of the journal [4] and, over the years, advocated for openness via the research articles published in the journal (for example, [5,6]). *PLOS Pathogens*’ position on Open Science also means it is already compliant with established and emerging funder policies around Open Science, such as Plan S [7] and the OSTP memo [8], that will bring United States–funded research in line with European Union and United Kingdom funders and further the adoption of Open Science.

Beyond *PLOS Pathogens*, we have recently seen the critical importance of Open Science practices including the sharing of preprints and the genomic sequencing data of SARS-CoV-2 to drive rapid innovation in research during the global response to the COVID-19 pandemic [9,10]. The AlphaFold Protein Structure Database [11] has over 350,000 protein models available, as well as the tools to view and interrogate the structure, that is available with an Open Access license. This enables and accelerates research into protein structures, for example, in response to the emergence of SARS-CoV-2, as well as in other areas related to pathogens research.

Funders and major journals require that data, such as proteomics and transcriptomics data, as well as new computer code are deposited in public repositories. The vast array and public availability of these primary datasets, as well as the stabilization of analysis platforms and methodology, permits retrospective meta-analysis of the wealth of useful data produced in different laboratories. For example, meta-analyses of many previously published and publicly available datasets from diverse tissues and cell lineages can be used to identify clusters of genes that are correlated in their expression across large datasets, which, in turn, can give clear insights into the transcriptional networks underlying specific tissue or cells (hematopoietic, mesenchymal lineages, etc.) or cellular functions (phagocytosis, cell-cycle, etc.). The resulting dataset can, in turn, be shared (for example, [12]) and used to look at numerous research questions. For example, by identifying novel intervention strategies to reverse the effects of immunosenescence on antigen-specific immunity in the skin of elderly humans in vivo [13], to help understand the transcriptional landscapes of thyroid cancer [14] and hepatocellular carcinoma [15], and to compare the cellular composition of whole blood samples from pediatric septic shock patients based on analysis of mRNA expression profiles [16].

## Cooperative and cocreative Open Science

The pathogens community has, and continues to, demonstrate its commitment to cooperative Open Science to improve and enhance science. One example would be the many community-curated virus databases used in pathogens research (see [17] for a review). A number of organizations, including the NIH HIV Reagent Program [18], the Biodefense & Emerging Infections Resources [19], the International Reagent Resource [20], the National Institute for Biological Standards and Control [21], and International Human Papillomavirus Reference Center [22], all freely provide reagents and standards that are vital to the pursuit of pathogen-focused research and have been provided by researchers in the field. Without this open sharing, research would be limited as it is reliant on the availability of high-quality and authenticated research materials. Similarly, Addgene [23], a nonprofit plasmid repository, facilitates the storage and distribution of plasmids across the globe that have been deposited by researchers for use by others. However, fees borne by the reuser, such as shipping costs, still act as a barrier to truly equitable Open Science. There is an opportunity here for the pathogens community to demonstrate their commitment further by creating pathways for researchers who still face barriers relating to the cost of research.

Cocreation is central to PLOS’ approach to Open Science. Open Science solutions will be most effective when they are initiated by the community and work with established practices and community norms. *PLOS Pathogens* embraced this approach with the adoption of the PLOS-wide mandatory data sharing policy in 2014, created after extensive consultation with the academic community [24]. Since 2014, the rate of data sharing, and data shared following best practice, has been increasing [25].

**Fig 2:**
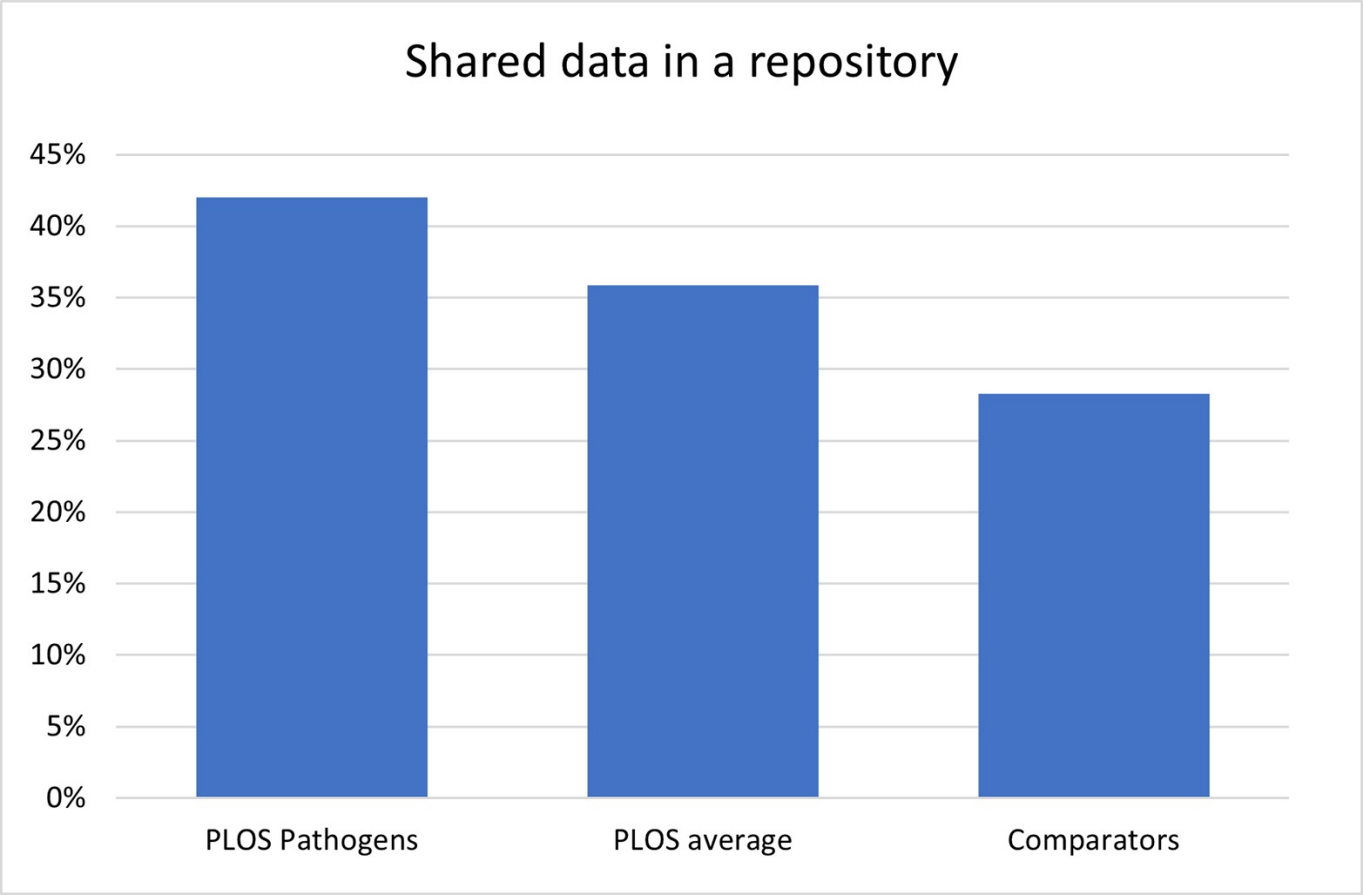
The percentage of shared data that is available from a repository for *PLOS Pathogens*, the average across PLOS journals and in comparator articles for research articles published between 1 Jan 2019 and 31 Mar 2023. Data from [27].

**Fig 3:**
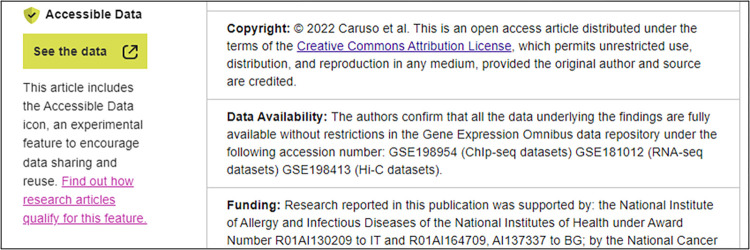
A snippet of a *PLOS Pathogens* article (https://doi.org/10.1371/journal.ppat.1010400), which now features the Accessible Data icon that takes readers straight to the data cited in the Data Availability statement.

We have worked with the community to offer experimental solutions we think have potential and to learn about why particular solutions do (or do not) work. PLOS can now monitor the effect of Open Science practices of authors, thanks to the Open Science Indicators initiative [26]. For example, we know that 48% of *PLOS Pathogens* authors sharing data use a repository compared to only 28% of authors in the broader scholarly community (see Fig 2) [27], and we can use this data to drill down further and understand which repositories our authors use. This has helped shape the extension of one of our new experimental features—the Accessible Data icon (see Fig 3)—to include the most commonly used repositories at PLOS, including Dryad, Figshare, Open Science Framework (OSF), Github, Zenodo, Gene Expression Omnibus, Sequence Read Archive, BioProject, and Demographic and Health Surveys [28]. Following the extension of the Accessible Data icon to include more repositories, the number of *PLOS Pathogens* articles that feature the Accessible Data icon has increased by almost 20 times. Results from the experiment have shown that datasets connected to articles carrying the Accessible Data feature have had significantly more engagement since the addition of the feature. In addition, qualitative results from the experiments support the potential of the icon to normalise data sharing and influence author behaviour [28].

Open, high-quality, peer-reviewed science is, and has always been, core to the mission of *PLOS Pathogens*, and we hope the journal can continue to work with the community to advance Open Science for years to come.
